# Cumulative ecological risk and cyberbullying among college students: a chain mediation model

**DOI:** 10.3389/fpsyg.2024.1302200

**Published:** 2024-02-26

**Authors:** Ruikai Miao, Zhuoyang Li

**Affiliations:** Mental Health Education and Guidance Center, Shijiazhuang Tiedao University, Shijiazhuang, China

**Keywords:** cumulative ecological risk, cyberbullying, belief in a just world, moral disengagement, college students

## Abstract

**Introduction:**

Cyberbullying among college students has been receiving increased research attention. Previous studies have focused primarily on the impact of a single risk factor on cyberbullying among college students. However, individual behavior is influenced by multiple ecosystems simultaneously, including family, school, and peers. To explore the effects of a single risk factor alone is not in line with the reality of everyday life, and the effect of the single risk factor can often be overestimated. Therefore, this study aimed to explore the impact of multiple risk factors, namely cumulative ecological risk, on cyberbullying, while analyzing the mediating roles of belief in a just world and moral disengagement.

**Methods:**

A survey was conducted among 805 college students from two universities in Hebei Province, China, using the cumulative ecological risk questionnaire, the cyberbullying scale, the belief in a just world scale, and the moral disengagement scale.

**Results:**

The results showed that: (a) Cumulative ecological risk was positively correlated with moral disengagement and cyberbullying, and negatively correlated with belief in a just world. Belief in a just world was negatively correlated with moral disengagement and cyberbullying. Moral disengagement was positively correlated with cyberbullying; (b) Belief in a just world partially mediated the relationship between cumulative ecological risk and cyberbullying; (c) Moral disengagement partially mediated the relationship between cumulative ecological risk and cyberbullying; (d) Belief in a just world and moral disengagement played a chain mediating role between cumulative ecological risk and college students’ cyberbullying.

**Discussion:**

This study provides valuable insight for the reduction of cyberbullying behavior among college students, and offers suggestions on how to create a more favorable online environment.

## 1 Introduction

Cyberbullying refers to the deliberate and repetitive use of online media by individuals or groups to engage in various forms of aggressive behavior, such as threats, insults, and harassment, toward others (Olweus and Limber, 2018). As one of the most active user groups on the Internet, college students have also become a high-risk group for cyberbullying. A survey conducted among college students in China showed that 39.18% of them had participated in cyberbullying (Zhu et al., 2016). Cyberbullying significantly impacts the physical and mental health of both the perpetrators and victims, and cyberbullies are prone to developing aggressive personalities and violent tendencies (Geel et al., 2017; Wang et al., 2020a). Meanwhile, victims of cyberbullying often experience psychological issues including anxiety, depression, or social phobia, and in extreme cases it can lead to extreme outcomes such as suicide (Shi et al., 2020). Given the high prevalence and serious consequences of cyberbullying among college students, it is extremely important to delve into the risk factors and underlying mechanisms that affect cyberbullying in this demographic. Based on ecosystem theory, this study examined the impact of the accumulation of risk factors in multiple domains such as family, school, and peers on cyberbullying among college students. Simultaneously, it also investigated the mediating roles of belief in a just world and moral disengagement in the relationship between cumulative ecological risk and cyberbullying. This study aims to systematically elucidate the mechanism of college students’ cyberbullying, and offer suggestions for reducing cyberbullying behavior among college students and creating a positive online environment.

## 2 Literature review

### 2.1 Cumulative ecological risk and cyberbullying

The theory of frustration-aggression suggests that risk factors in domains such as family, school, or peer groups can lead to feelings of frustration in individuals, which can in turn manifest as aggressive behaviors, including bullying (Gilbert and Bushman, 2020). As cyberbullying represents an online extension of bullying behavior, numerous empirical studies have found that risk factors in domains such as family, school, and peers are key precipitating factors for individuals engaging in cyberbullying. However, these studies tend to focus on the impact of single or a few risk factors on cyberbullying.

First of all, family risk factors are pivotal in precipitating cyberbullying among college students. A deteriorated family environment increases the probability of being an aggressor of cyberbullying, whereas a favorable family environment decreases this probability (Martinez-Monteagudo et al., 2018). The development of psychology and behavior among college students is not yet fully mature. Students with poor parent–child relationship and a lack of familial support often experience more loneliness. They tend to alleviate internal pressures and dissatisfaction with reality through the outlet of cyberbullying (Safaria and Suyono, 2020; Wang et al., 2020b). In addition, students with lower parental education and lower socioeconomic status have more negative emotions, increasing the likelihood of cyberbullying (Liu et al., 2021).

Secondly, besides family, school is the primary living space for college students. Therefore, the role of school risk factors in cyberbullying should not be underestimated. Studies have found that classmate relationships can significantly predict adolescent cyberbullying, and bad classmate relationships are a risk factor for cyberbullying (Gao et al., 2020; Wang et al., 2021). Furthermore, when students have a lower degree of connection to the school (it refers to the degree to which students feel respect, care, and the sense of belonging in school.), they are more likely to engage in cyberbullying behaviors (Bevilacqua et al., 2016; Law et al., 2022).

Finally, peer risk factors are also key factors affecting cyberbullying among college students. Social learning theory posits that individuals are inclined to exhibit behaviors similar to those of their peers through the processes of observation and imitation (Bandura, 1977). Yang et al. (2021) examined the association between deviant peer affiliation and adolescent cyberbullying, and found that adolescents who reported higher deviant peer affiliation were more likely to bully others online. In addition, without the support of friends, individuals are susceptible to negative emotions and then they will vent their emotions through cyberbullying (Li, 2022).

In general, previous studies have primarily focused on the impact of single risk factors on cyberbullying, with limited exploration into the cumulative effects of multi-domain risk factors on cyberbullying among college students. Ecosystem theory posits that individual development is influenced by multiple ecological subsystems simultaneously, such as family, school, and peers (Bronfenbrenner, 1979). In other words, individuals often face risks across multiple domains simultaneously, and examining the effects of only one risk factor will not reflect the complex realities of individuals’ everyday lives, and may lead to an overestimation of the impact of that one risk factor (Evans et al., 2013). Therefore, in recent years, researchers have begun to investigate the cumulative effects of risk factors on individual development, such as internet addiction, academic achievement, mental health (Li et al., 2016; Tan et al., 2021; Miao et al., 2023). With this in mind, the present study aimed to explore the influence of cumulative ecological risks – encompassing risk factors from multiple domains – on cyberbullying among college students.

### 2.2 Mediating roles of belief in a just world and moral disengagement

Belief in a just world refers to the belief of individuals that they live in a fair world, where people get what they deserve and deserve what they get (Lerner and Miller, 1978). Meanwhile, shattered assumption theory posits that risk factors can challenge individuals’ pre-existing stable perceptions of the world, leading to the formation of negative worldviews and a belief that the world is unjust (Janoff-Bulman, 2010). Research has shown that risk factors such as family economic pressure (Liu et al., 2020) and social exclusion (Chen, 2021) significantly and negatively predict one’s belief in a just world. The higher the family economic pressure and the more social exclusion one experiences, the lower one’s level of belief in a just world. Furthermore, again according to shattered assumption theory, individuals often engage in deviant behaviors such as bullying as a way to restore cognitive balance in response to their perception of an unjust world. Donat et al. (2023) and colleagues conducted a survey to explore the relationship between university students’ belief in a just world and cyberbullying, and revealed that belief in a just world is a significant predictor of cyberbullying, with lower levels of belief in a just world associated with higher frequencies of cyberbullying occurrence. Therefore, the current study hypothesized that belief in a just world plays an important mediating role in the relationship between cumulative ecological risks and cyberbullying among college students.

Moral disengagement refers to a cognitive tendency exhibited by individuals, characterized by redefining one’s own behavior to minimize harm, reduce personal responsibility for the consequences, and decrease empathy toward the victims (Bandura et al., 2017). Moral disengagement is a significant cognitive factor contributing to unethical behavior, whereby individuals rationalize their unethical actions as a means to alleviate their inner guilt. Studies have indicated that moral disengagement significantly predicts unethical behaviors such as cyberbullying among college students (Zeng and Xue, 2002; Fu et al., 2020; Hu and Xiong, 2024). The higher the degree of moral disengagement, the greater the frequency of cyberbullying behavior. Furthermore, as a cognitive component within the moral domain, moral disengagement is influenced by external environmental factors. Studies have found that multiple domains of risk factors, including family and community, collectively contribute to moral disengagement in that increased exposure to risk factors leads to lower moral identification and significantly higher levels of moral disengagement (Hyde et al., 2010). Therefore, the current study hypothesized that moral disengagement plays a crucial mediating role between cumulative ecological risks and cyberbullying among college students.

Belief in a just world and moral disengagement are both important predictors of cyberbullying, and research has shown a close relationship between the two. Belief in a just world is significantly negatively correlated with moral disengagement, indicating that individuals with lower belief in a just world tend to exhibit higher levels of moral disengagement (Zhang, 2022). Risk factors can impair one’s belief in a just world, and perceptions and experiences of unfairness can impact their identification with social moral norms. In these circumstances, individuals will often break their own moral standards and activate the mechanism of moral disengagement to engage in unethical behaviors (Gini et al., 2013). With this in mind, the current study hypothesized that belief in a just world and moral disengagement play a chain mediating role between cumulative ecological risk and cyberbullying among college students.

## 3 Methods

### 3.1 Participants

A random cluster sampling method was used to select college students from two universities in Hebei Province, China, as participants in the study. One is a science and engineering university, and the other is a humanities and social science university. Before completing the survey, the students were assured that the survey data would be used exclusively by the research team, and would not be accessed by other personnel. The data collection took place during a regularly-scheduled class, and after obtaining the students’ informed consent, the questionnaires were administered by the teaching faculty. A total of 878 questionnaires were distributed, and 805 valid questionnaires were obtained, resulting in an effective rate of 91.7%. Among the respondents, 458 students were male (56.9%) and 347 were female (43.1%). Furthermore, 282 were freshmen (35.0%), 313 were sophomores (38.9%), 128 were juniors (15.9%), and 82 were seniors (10.2%), and the average age of respondents was 19.89 years. This study was approved by the academic committee of the researchers’ institution of affiliation.

### 3.2 Tools

#### 3.2.1 Cumulative ecological risk

In theory, all ecological factors can be included in the measurement of cumulative ecological risk. However, in terms of the necessity and feasibility of research, it is advisable and even essential to consider only the significant risk factors closely related to developmental outcomes. Therefore, based on ecosystem theory and considering previous research on cumulative ecological risk and cyberbullying, the following nine representative risk factors were selected from the family, school, and peer subsystems to construct the cumulative ecological risk index used in this study:

1. Parental education level: Two items were used to measure the educational levels of the respondents’ father and mother separately, each rated using a six-point scale, from 1 (primary school or below) to 6 (postgraduate or higher). If either parent had a high school education or below (including vocational schools and technical colleges), the response was coded as 1, indicating risk; otherwise, it was coded as 0, indicating no risk.
2. Family type: Following Dong and Lin (2011), a single item was used to measure family type: “Who are the family members you currently live with?” If the respondent selected the option indicating they do not live with their biological parents, the response was coded as 1, indicating risk; otherwise, it was coded as 0 indicating no risk.
3. Family socioeconomic status: Following Xu et al. (2012), a single item was used to measure family socioeconomic status: “Compared to other students in your school, how do you perceive the social status of your family?” The item was rated using a five-point scale ranging from 1 (significantly lower level than average) to 5 (significantly higher level than average). If the score was lower than the average level, it was coded as 1, indicating risk; otherwise, it was coded as 0, indicating no risk.
4. Parent–child relationship: The revised Parent–Child Closeness Scale as developed by Zhang et al. (2006) was used to assess respondents’ parent–child relationships. The scale consists of 10 items, each rated on a five-point scale ranging from 1 (almost never) to 5 (almost always). Higher scores indicate a better parent–child relationship. In this study, the Cronbach’s α coefficient for this scale was 0.78.
5. Family support: The Family Support subscale of the Perceived Social Support Scale, as developed by Jiang (1991), was used. The subscale consists of four items, with each one rated on a seven-point scale ranging from 1 (strongly disagree) to 7 (strongly agree). Higher scores indicate the respondent experiences a greater level of family support. In this study, the Cronbach’s α coefficient for this scale was 0.93.
6. School connectedness: The School Connectedness Scale as developed by Resnick et al. (1997) was used, consisting of six items rated on a five-point scale ranging from 1 (strongly disagree) to 5 (strongly agree). Higher scores indicate a higher degree of school connectedness. In this study, the Cronbach’s α coefficient for this scale was 0.93.
7. Classmate relationships: The Classmate Relationships subscale of the Interpersonal Relationships Scale as developed by Wang (2013) was utilized. The subscale is made up of three items, each of which is rated on a four-point scale, ranging from 1 (not at all consistent) to 4 (very consistent). Higher scores indicate better classmate relationships. In this study, the Cronbach’s α coefficient for this scale was 0.88.
8. Friendship support: The Friendship Support subscale of the Perceived Social Support Scale, as developed by Jiang (1991), was utilized. The subscale consists of three items, each rated on a seven-point scale ranging from 1 (strongly disagree) to 7 (strongly agree). Higher scores indicate a higher level of friendship support. In this study, the Cronbach’s α coefficient for this scale was 0.96.
9. Deviant peer affiliation: The Deviant Peer Affiliation Questionnaire as developed by Li et al. (2013) was employed to assess the level of engagement the respondent experiences in deviant peer affiliation. The questionnaire consists of eight items, each of which is rated on a five-point scale ranging from 1 (none) to 5 (all). Higher scores indicate the respondent has a greater number of deviant peers. In this study, the Cronbach’s α coefficient for this scale was 0.90.

In scales 4 to 8 (as noted above), a score equal to or below the 25th percentile was coded as 1, indicating risk, while those above the 25th percentile were coded as 0, indicating no risk. For the 9th scale, a score equal to or above the 75th percentile was coded as 0, indicating no risk, while the rest were coded as 1, indicating risk. Finally, the cumulative ecological risk index was obtained by summing up the scores of all measured risk factors. In this study, The Cronbach’s α coefficient of the total questionnaire was 0.88.

#### 3.2.2 Cyberbullying

The scale measuring cyberbullying utilized in this study was developed initially by Erdur-Baker and Kavsut (2007), and subsequently revised by Zhou et al. (2013). The scale comprises a total of 18 items, some examples of which include: “Spread rumors about someone on the Internet” and “Send harmful text messages to someone.” Each item is rated on a four-point scale ranging from 1 (never) to 4 (5 or more times). Higher scores indicate a greater frequency of engaging in cyberbullying behaviors. In the current study, the scale demonstrated excellent internal consistency, with a Cronbach’s α coefficient of 0.95.

#### 3.2.3 Belief in a just world

The Belief in a Just World Scale used in this study was compiled by Dalbert (1999) and translated and revised by Su et al. (2012). The scale consists of 13 items, some examples of which include “I believe that, by and large, people get what they deserve” and “I think people try to be fair when making important decisions.” Each item is rated using a six-point Likert scale, ranging from 1 (strongly disagree) to 6 (strongly agree). Higher scores indicate a stronger belief in a just world. The scale demonstrated excellent reliability in the current study, with a Cronbach’s α coefficient of 0.96.

#### 3.2.4 Moral disengagement

The Moral Disengagement Scale developed by Bandura et al. (1996) and revised by Wang and Yang (2010) was used in this study. The scale comprises 26 items, some examples of which include: “It is alright to fight to protect your friends” and “It is alright to beat someone up who badmouths your family.” Each item is rated using a five-point Likert scale, ranging from 1 (completely disagree) to 5 (completely agree). The higher the score, the higher the respondent’s level of moral disengagement. The scale exhibited high internal consistency in the current study, with a Cronbach’s α coefficient of 0.94, indicating strong consistency among the items.

### 3.3 Data analysis

Data analysis was conducted using SPSS 24.0. Descriptive statistics were employed to calculate the mean and standard deviation of each variable. Correlation analysis was performed to explore the relationships among cumulative ecological risk, belief in a just world, moral disengagement, and cyberbullying. The mediating effect of belief in a just world and moral disengagement was examined using the SPSS Process plugin.

## 4 Results

### 4.1 Test of common method bias

To mitigate the potential issue of common method bias associated with self-report questionnaires, appropriate measures were taken during the survey administration, following recommendations from previous studies (Zhou and Long, 2004) including ensuring the anonymity of questionnaire responses and providing standardized instructions to all respondents. After the completion of the data collection, Harman’s single-factor test was conducted to assess the presence of common method bias. The results indicated that there were 16 factors with eigenvalues greater than 1, however, the first factor accounted for only 23.28% of the variance, which is below the critical threshold of 40%. This suggested that there was no significant common method bias in this study.

### 4.2 Describe statistics and correlation analysis

Pearson’s correlation coefficient was used to test the relationships among cumulative ecological risk, belief in a just world, moral disengagement, and cyberbullying. As shown in Table 1, there were significant positive correlations observed between cumulative ecological risk and cyberbullying, as well as between cumulative ecological risk and moral disengagement. Conversely, belief in a just world demonstrated significant negative correlations with cumulative ecological risk, moral disengagement, and cyberbullying.

**Table 1:** Describe statistics and correlation analysis.

	M	SD	1	2	3	4
Cumulative ecological risk	2.24	1.13	1			
Cyberbullying	1.07	0.30	0.24^***^	1		
Belief in a just world	4.13	0.91	−0.28^***^	−0.29^***^	1	
Moral disengagement	2.17	0.70	0.26^***^	0.34^***^	−0.29^***^	1

*p < 0.05, **p < 0.01, ***p < 0.001.

### 4.3 Mediating effects test

The mediating effects of belief in a just world and moral disengagement between cumulative ecological risk and cyberbullying were examined using Model 6 in the SPSS program PROCESS as developed by Hayes. The results of regression analysis (see Table 2) revealed that, controlling for gender and grade, cumulative ecological risk significantly positively predicted cyberbullying among college students (*β* = 0.07, *p* < 0.001, 95% CI: 0.05 ~ 0.08). After incorporating belief in a just world and moral disengagement into the regression equation, cumulative ecological risk significantly negatively predicted belief in a just world (*β* = −0.22, *p* < 0.001, 95% CI: −0.28 ~ −0.17) and significantly positively predicted moral disengagement (*β* = 0.11, *p* < 0.001, 95% CI: 0.07 ~ 0.15). Belief in a just world significantly negatively predicted moral disengagement (*β* = −0.18, *p* < 0.001, 95% CI: −0.23 ~ −0.13) and significantly negatively predicted cyberbullying (*β* = −0.06, *p* < 0.001, 95% CI: −0.08 ~ −0.04). Moral disengagement significantly positively predicted cyberbullying (*β* = 0.12, *p* < 0.001, 95% CI: 0.09 ~ 0.14). Overall, cumulative ecological risk still significantly positively predicted cyberbullying (*β* = 0.04, *p* < 0.01, 95% CI: 0.02 ~ 0.05).

**Table 2:** Regression analysis of the relationship between various variables.

Regression equation		Overall fit index		Significance of regression coefficient		
Dependent variables	Independent variables	*R*	*R^2^*	*F*	*β*	*t*
Cyberbullying		0.25	0.06	18.05^***^		
	Gender				0.03	1.69
	Grade				−0.01	−1.14
	Cumulative ecological risk				0.07	7.22^***^
Belief in a just world		0.28	0.08	22.32^***^		
	Gender				−0.01	−0.08
	Grade				0.03	1.17
	Cumulative ecological risk				−0.22	−8.07^***^
Moral disengagement		0.38	0.14	33.52^***^		
	Gender				−0.20	−4.12^***^
	Grade				0.05	2.12^*^
	Cumulative ecological risk				0.11	5.21^***^
	Belief in a just world				−0.18	−6.90^***^
Cyberbullying		0.42	0.18	35.21^***^		
	Gender				0.05	2.90^**^
	Grade				−0.02	−1.51
	Cumulative ecological risk				0.04	3.91^**^
	Belief in a just world				−0.06	−5.14^***^
	Moral disengagement				0.12	7.89^***^

*p < 0.05, **p < 0.01, ***p < 0.001.

**Figure 1:**
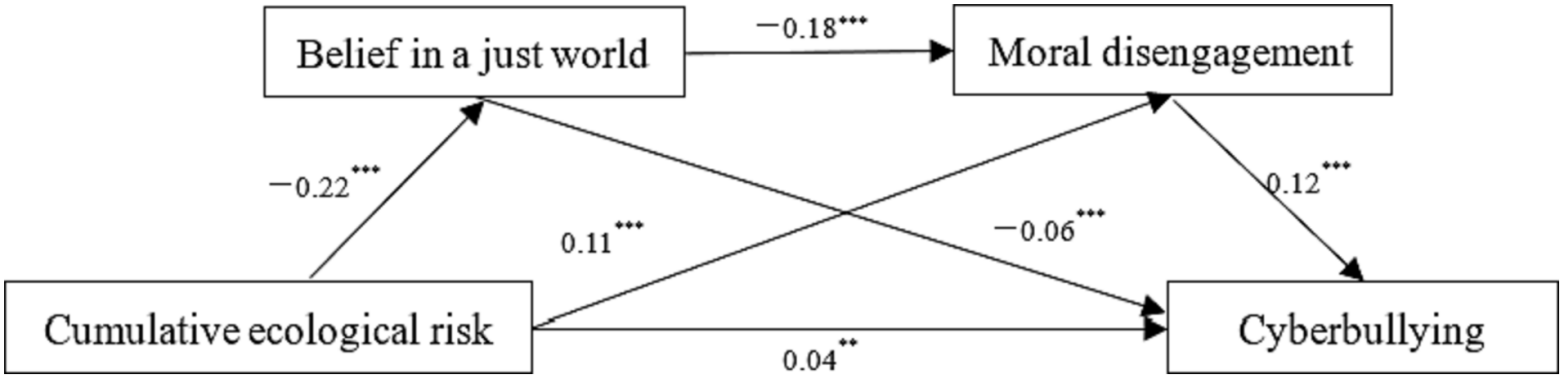
The chain mediating effects of belief in a just world and moral disengagement. **p < 0.01, ***p < 0.001.

The results of the mediating effects test (see Table 3) indicate that the mediating effects of belief in a just world and moral disengagement, as well as the chain mediating effects of belief in a just world and moral disengagement are all significant, with 95% confidence intervals which do not include zero. Specifically, the mediating effects consist of three pathways: (a) cumulative ecological risk → belief in a just world → cyberbullying, the effect value was 0.0128, accounting for 19.63% of the total effect value; (b) cumulative ecological risk →moral disengagement→ cyberbullying, the effect value was 0.0128, accounting for 19.63% of the total effect value; (c) cumulative ecological risk → belief in a just world→ moral disengagement →cyberbullying, the effect value was 0.0046, accounting for 7.06% of the total effect value (Figure 1).

**Table 3:** Results of the mediating effects test.

	Effect size	Standard error	Bootstrap lower limit	Bootstrap upper limit
Total indirect effect	0.0302	0.0098	0.0144	0.0545
Cumulative ecological risk → Belief in a just world → Cyberbullying	0.0128	0.0058	0.0031	0.0253
Cumulative ecological risk → Belief in a just world→ Moral disengagement →Cyberbullying	0.0046	0.0022	0.0015	0.0108
Cumulative ecological risk →Moral disengagement→ Cyberbullying	0.0128	0.0066	0.0039	0.0300

## 5 Discussion

Previous studies on cyberbullying have paid less attention to the impact of multiple ecological risk factors. Based on ecosystem theory, this study selected representative risk factors from the domains of family, school, and peers to investigate the effects and underlying mechanisms of cumulative ecological risk on cyberbullying among college students. Meaningful findings were obtained through this exploration.

The analysis results showed that cumulative ecological risk significantly and positively predicts cyberbullying among college students, indicating that the more cumulative ecological risk factors one experiences, the higher their likelihood of engaging in cyberbullying behaviors. This finding is consistent with that of previous research on the impact of cumulative ecological risk on online deviant behavior (Li et al., 2016; Guan et al., 2023). Support from family, school, and peers is crucial for the healthy development of college students. However, if these crucial domains are filled with the existence of multiple risk factors, such as low family socioeconomic status, weak school connectedness, and limited support from family and friends, individuals may experience significant frustration and be prone to increased negative emotions such as anger and depression (Tan et al., 2020). Studies have found a significant positive correlation between cumulative ecological risk and negative emotions among adolescents (Xiong et al., 2020; Miao et al., 2023). In such circumstances, the anonymity offered by the Internet can serve as an outlet for college students to vent their negative emotions, leading to the occurrence of cyberbullying. In addition, the psychological self of college students is not mature, and their psychology and behavior are greatly influenced by peers. College students who are exposed to risks in multiple domains often lack guidance and supervision from parents and teachers in their lives (Tan et al., 2023). This lack of guidance makes them susceptible to forming associations with deviant peers, leading to a higher likelihood of engaging in cyberbullying behaviors. The results of the mediation analysis indicated that belief in a just world plays a mediating role between cumulative ecological risk and cyberbullying, supporting shattered assumption theory. College students may face risk factors such as poor parent–child relationships, low school connectedness, or a lack of friend support. As these risk factors accumulate, individuals’ experiences of unfairness in their environment are enhanced, which leads to a compromised belief in world justice and the development of negative cognitive perceptions of an “unjust” world (Su et al., 2013). The absence of a belief in a just world has a significant negative impact on individuals’ social cognition and adaptation. Ucar et al. (2019) explored the relationship between belief in a just world and life satisfaction. Belief in a just world can increase college students’ sense of control, which in turn increases life satisfaction. However, belief in an unjust world can reduce college students’ sense of control over external events, and is often accompanied by negative emotions such as anxiety or anger, thereby reducing life satisfaction. In response to these emotional grievances, when using the Internet, individuals may seek to exert dominance over others by engaging in cyberbullying, attempting to compensate for their own lack of control (Guo, 2021).

The analysis results also showed that moral disengagement mediates the relationship between cumulative ecological risk and cyberbullying. According to social learning theory, family, school, and peers are all important sources for individuals in their formation of moral cognition (Jin, 2020). However, individuals who experience multiple risks in these domains are prone to develop moral cognitive distortions, leading to a weakening of their self-regulatory mechanisms for moral adjustment and an elevation in their level of moral disengagement. Studies have found that individuals with negative parental upbringing and who associate with delinquent peers exhibit significantly higher levels of moral disengagement (Li, 2019). When using the Internet, these individuals tend to interpret others’ words and actions in a negative and hostile manner, exhibiting more aggressiveness. Moral disengagement, as a specific cognitive mechanism, enables individuals to rationalize their unethical deviant behaviors, thereby reducing their associated feelings of guilt and ultimately leading to the occurrence of cyberbullying (Maria et al., 2020; Wang et al., 2022).

The results of the mediation analysis also indicate that cumulative ecological risk can influence college students’ engagement in cyberbullying through the chain-mediated effects of belief in a just world and moral disengagement. The more risks faced by college students, the more likely they are to perceive themselves as living in an unfair environment, which subsequently influences the development of their belief in justice. Research has found that individuals with low belief in a just world tend to have suppressed moral identification, leading to changes in their cognitive processes that make them more prone to violating moral standards and engaging in moral disengagement (Li et al., 2018; Gao et al., 2022). As a result, these individuals are more likely to engage in cyberbullying behavior, even though it may harm others, because it aligns with their internal moral logic.

## 6 Limitations and future prospects

This study explores the relationship between cumulative ecological risk and college students’ cyberbullying. By constructing a chain mediation model, it reveals the internal mechanism of cumulative ecological risk on cyberbullying which has both important theoretical and practical values for understanding college Students’ cyberbullying. It also provides a premise for further research on how to prevent and address cyberbullying among college Students. However, there are still three shortcomings in the research: Firstly, although the risk factors selected in this study are typical, not all potential risk factors have been included. Future research can test the findings of this study by incorporating as many risk factors as possible. Secondly, this study only explored the mediating roles of belief in a just world and moral disengagement. Studies have found a significant correlation between cumulative ecological risk and self-control (Tan et al., 2022). Moreover, self-control is a crucial internal factor in predicting cyberbullying (Savage and Tokunaga, 2017). Therefore, future research can further analyze the underlying mechanisms of cumulative ecological risks affecting cyberbullying from the perspective of self-control. Thirdly, the research mainly used questionnaire survey data, but the data obtained from the questionnaire survey is difficult to demonstrate the complex process of individual and environmental interaction. In the future, Agent-Based Model (ABM) methods (Tseng et al., 2014; Qiu, 2022) can be used to further explore the characteristics of various variables that affect the evolution of college students’ cyberbullying and cannot be accurately analyzed by the questionnaire survey, such as the intensity and duration of risks, in order to find the best intervention strategy for college students’ cyberbullying.

## 7 Conclusion

Cumulative ecological risk has a direct and significant impact on college students’ cyberbullying. The more ecological risk factors in the domains of family, school, and peers, the more likely college students are to engage in cyberbullying. Moreover, belief in a just world and moral disengagement are found to be important mediators in the relationship between cumulative ecological risk and cyberbullying. Specifically, three distinct mediating paths emerge: the separate mediating role of belief in a just world, the separate mediating role of moral disengagement, and the chain mediating roles of belief in a just world and moral disengagement. Therefore, to address the issue of cyberbullying, families, schools, and society should work together to reduce the multi-field risk factors faced by college students. Meanwhile, enhancing the belief in a just world and reducing moral disengagement are also key measures to intervene in cyberbullying among college students.

## Data availability statement

The raw data supporting the conclusions of this article will be made available by the authors, without undue reservation.

## Ethics statement

The studies involving humans were approved by Academic Committee of the School of Marxism, Shijiazhuang Tiedao University. The studies were conducted in accordance with the local legislation and institutional requirements. The participants provided their written informed consent to participate in this study.

## Author contributions

RM: Writing – original draft, Writing – review & editing. ZL: Data curation, Writing – review & editing.
